# Editorial: Prodromal Parkinson's Disease

**DOI:** 10.3389/fneur.2020.634490

**Published:** 2021-01-12

**Authors:** David Crosiers, Patrick Santens, K. Ray Chaudhuri

**Affiliations:** ^1^Department of Neurology, Antwerp University Hospital, Edegem, Belgium; ^2^Translational Neurosciences, Faculty of Medicine and Health Sciences, Institute Born-Bunge, University of Antwerp, Antwerp, Belgium; ^3^Department of Neurology, Ghent University Hospital, Ghent, Belgium; ^4^Parkinson Foundation International Centre of Excellence, King's College Hospital and King's College, London, United Kingdom

**Keywords:** Parkinson's disease, prodromal, REM sleep behavior disorder, biomarkers, neuroprotection

In the prodromal stage of Parkinson's disease (PD), cardinal motor symptoms (bradykinesia, rigidity, and resting tremor) have not yet developed and hence clinical diagnostic criteria for PD are not met (1, 2). However, neurodegeneration is already ongoing, and a variety of motor and non-motor symptoms may be identified. In addition to these clinical prodromal markers, recent studies have demonstrated a potentially important role of imaging, biofluid, and tissue markers in the characterization and identification of the prodromal stage of PD (3). An extensive and up-to-date overview of the status of these prodromal markers was included in the recent publication of the MDS research criteria for prodromal PD (2).

In this special issue of Frontiers in Neurology – Movement Disorders, a total of seven contributions add to the clinical and non-clinical aspects of prodromal PD, each targeting specific issues that need further exploration, but sometimes promising as potential biomarkers in the identification of prodromal PD or as treatment targets to tackle disease progression. Hustad and Aasly provide an overview of clinical, fluid, tissue, genetic, and imaging markers of prodromal PD. Clinical prodromal markers can include non-motor symptoms (hyposmia, REM sleep behavior disorder, constipation, excessive daytime sleepiness, depression, cognitive symptoms, autonomic nervous system dysfunction) of varying likelihood ratio to predict PD. Subtle motor signs in prodromal PD can be observed by the clinician or by using quantitative motor testing, however future wearable technologies will probably contribute to more detailed insights in these motor markers. Voice changes such as modulations in volume, pitch and tone can be detected in the early stage of PD, but it is still not entirely clear how vocal abnormalities could be used as a prodromal marker (4, 5). Similarly, alterations in auditory processing have been described in early stage PD, including difficulties with tone discrimination, and with perception of loudness and emotional aspects of speech. De Groote et al. provide a literature review of central auditory processing in early PD and point out which audiological and electrophysiological techniques could be explored to identify auditory prodromal markers.

The loss of muscle atonia during rapid-eye movement sleep (REM sleep without atonia or RSWA) and dream-enactment behaviors are the two characteristic features of REM sleep behavior disorder (RBD) (6). Idiopathic RBD is a highly specific marker for future development of a synucleinopathy (7, 8). Roguski et al. discuss the epidemiology, etiology, diagnostic considerations, and management of (idiopathic) RBD in a narrative literature review. The authors also provide ethical reflections and practical suggestions to guide the clinician in the difficult discussion of the prognosis of idiopathic RBD to asymptomatic, or potentially prodromal PD patients. Providing sufficient information, counseling and offering clinical follow-up visits to these individuals, without overloading them with extensive details about the possible risks, remains a balancing act for the clinician and researcher (9).

Different imaging modalities can contribute to define imaging biomarkers for prodromal PD, including single-photon-emission computed tomography, positron-emission tomography, magnetic resonance imaging (MRI) (10). Ellmore et al. have performed resting-stage functional MRI (fMRI) in RBD patients, PD patients, and control individuals to explore the substantia nigra functional connectivity and its relation to the serum uric acid levels. Lower levels of serum uric acid, are indeed associated with higher risk for PD in men. Catecholamine biofluid markers are interesting candidate markers for prodromal PD, given the noradrenergic, serotonergic and dopaminergic dysfunctions in prodromal and early PD as well as the recent “body first” vs. “brain first” concept of PD pathogenesis. Vermeiren et al. have reviewed the available evidence for the role of catecholamine biofluid markers in prodromal PD and provide recommendations for further exploration of extracellular vesicles as a potential novel biomarker.

Asymptomatic individuals carrying the G2019S mutation in *LRRK2* are at-risk to develop PD, with disease penetrance ranging from 25 to 42.5% by the age of 80 years, with a variable expression of non-motor symptoms including sleep dysfunction (11, 12). Longitudinal follow-up of these asymptomatic mutation carriers is helpful for defining the early stages of prodromal PD, as in a proportion of these individuals a phenoconversion to PD will be observed. Crown et al. have investigated potential prodromal features in a LRRK2-G2019S knock-in mouse model. The LRRK2 kinase inhibitor MLi-2 was administered in a subgroup of the mice. The results of this study, comparing the wild-type and G2019S knock-in populations, suggest alterations in behavioral and physiological aspects of sleep in the G2019S mice.

The ultimate goal of the accurate identification of individuals with prodromal PD is the administration of a neuroprotective treatment to these individuals to slow down or halt disease progression. Sportelli et al. discuss the association between diabetes mellitus type 2 and PD, and highlight the role of metformin as a potential disease-modifying treatment for PD. Preclinical evidence in animal models and clinical evidence in other age-related diseases suggest that metformin might delay aging-related symptoms and manifestations as well as offer anti-inflammatory activity.

Research in the field of prodromal PD is a fascinating and rapidly developing area, involving fundamental and translational as well as clinical and paraclinical aspects. Validation of biomarkers that allow to identify individuals at risk for developing clinical PD is a currently unmet need. Further developments in this area are urgently needed in order to manage the growing burden of PD.

## Author Contributions

DC, PS, and KRC wrote the first draft of the manuscript and critically revised the content of the manuscript. All authors contributed to the article and approved the submitted version.

## Conflict of Interest

The authors declare that the research was conducted in the absence of any commercial or financial relationships that could be construed as a potential conflict of interest.
